# Neuroprotective Effect of *Marrubium vulgare* Extract in Scopolamine-Induced Cognitive Impairment in Rats: Behavioral and Biochemical Approaches

**DOI:** 10.3390/biology13060426

**Published:** 2024-06-09

**Authors:** Maria Lazarova, Miroslava Stefanova, Petko Denev, Teodora Taseva, Valya Vassileva, Krasimira Tasheva

**Affiliations:** 1 Institute of Neurobiology, Bulgarian Academy of Science, 1113 Sofia, Bulgaria; mira_stefanova@mail.bg; 2 Laboratory of Biologically Active Substances, Institute of Organic Chemistry with Centre of Phytochemistry, Bulgarian Academy of Sciences, 4000 Plovdiv, Bulgaria; petko.denev@orgchm.bas.bg; 3 Institute of Plant Physiology and Genetics, Bulgarian Academy of Sciences, Acad. Georgi Bonchev Str., Block 21, 1113 Sofia, Bulgaria; tasevatk@abv.bg (T.T.); krasitasheva@abv.bg (K.T.)

**Keywords:** *Marrubium vulgare*, phytotherapy, dementia, cognitive enhancement, cholinergic pathways, biogenic amines, neurotrophic factors

## Abstract

**Simple Summary:**

Cognitive deficits, including spatial working and recognition memory impairment, are a common feature of Alzheimer’s disease with current therapies offering limited efficacy. *Marrubium vulgare*, a member of the Lamiaceae family, has shown potential to alleviate spatial memory impairment in a model of experimental dementia in rats through its antioxidant and acetylcholinesterase inhibitory activities. The aim of this study was to examine the effect of *M. vulgare* on recognition memory in healthy and dementia-affected rats after 21 days of oral administration. Memory performance was evaluated by the novel object recognition test. Levels of neurotransmitters acetylcholine, noradrenaline (NA), and serotonin, as well as the protein expression of the brain-derived neurotrophic factor (BDNF) and the phosphorylation of the cAMP response element-binding protein (p-CREB), were measured. The expression levels of *BDNF* and *CREB* were evaluated via RT-PCR in the cortex and hippocampus. Our result revealed that *M. vulgare* ameliorated recognition memory impairment in dementia rats by preserving cholinergic function in the hippocampus, increasing NA levels in the brain, and restoring pCREB expression in the cortex following their reduction in the experimental model used. In healthy rats, the extract upregulated the expression of *BDNF* and *pCREB* in the cortex. These findings suggest that *M. vulgare* has potential as a therapeutic agent for cognitive impairments in various neurodegenerative diseases.

**Abstract:**

The potential of *Marrubium vulgare* to alleviate scopolamine (Sco)-induced deficits in spatial working memory has drawn considerable scientific interest. This effect is partly attributed to its potent antioxidant and acetylcholinesterase inhibitory (AChEI) activities. This study examined the effects of *M. vulgare* extract, standardized to marrubiin content, on recognition memory in healthy and Sco-treated rats. Male Wistar rats (200–250 g) were divided into four groups. The extract was orally administered for 21 days and Sco (2 mg/kg) was intraperitoneally injected for 11 consecutive days. Memory performance was assessed using the novel object recognition test. Levels of acetylcholine (ACh), noradrenaline (NA), serotonin (Sero), and brain-derived neurotrophic factor (BDNF) and the phosphorylation of cAMP response element-binding protein (p-CREB) were evaluated in the cortex and hippocampus via ELISA. *BDNF* and *CREB* expression levels were assessed using RT-PCR. The results showed that *M. vulgare* significantly alleviated Sco-induced memory impairment, preserved cholinergic function in the hippocampus, increased NA levels in the brain, and restored pCREB expression in the cortex following Sco-induced reduction. In healthy rats, the extract upregulated *BDNF*, *pCREB,* and *Bcl2* expression. Our findings indicate that the neuroprotective effects of *M. vulgare* may be linked to the modulation of cholinergic function, regulation of NA neurotransmission, and influence on key memory-related molecules.

## 1. Introduction

Alzheimer’s disease (AD) stands out as one of the most common forms of dementia in the elderly population [1,2]. Dementia involves a decline in cognitive abilities due to the death of brain cells [3], which consequently impairs cognitive functions such as memory, reasoning, and learning capacity [4].

The cognitive impairment in AD is predominantly linked to reductions in cholinergic neurotransmission systems and neuronal dysfunction [5,6]. Decreased levels of brain acetylcholine (ACh), the overexpression of acetylcholinesterase (AChE) and glutamate excitotoxicity [7], decreased levels of the ACh synthesizing enzyme choline acetyltransferase (ChAT), the altered function of both muscarinic and nicotinic ACh receptors [5,8,9,10,11,12,13,14,15,16], and increased oxidative stress [17] are some of the main features of the disease.

Correspondingly, the use of cholinergic receptor antagonists such as scopolamine (Sco) is a widely used method for inducing cognitive impairment in rodents by blocking the cholinergic system [18]. This approach represents the most commonly used model of pharmacologically induced dementia [19,20]. Sco administration leads to changes in exploratory behavior, as well as spatial, associative, and recognition memory in experimental animals [20,21,22,23,24,25]. In addition, impairment in antioxidant defense systems, increased oxidative stress, mitochondrial dysfunction, apoptosis, and neuroinflammation have also been reported [26]. These neurochemical disturbances mainly affect the hippocampus and prefrontal cortex of experimental animals.

Currently, medications for Alzheimer’s disease primarily focus on symptom management, with AChE inhibitors being the most effective treatment. Four AChE inhibitors are approved and in use for AD treatment: donepezil, rivastigmine, tacrine, and galantamine [27,28,29,30,31]. These drugs alleviate cognitive deficits by increasing the bioavailability of acetylcholine in cholinergic synapses [32,33]. Despite their therapeutic benefits [34], they neither delay nor stop the progression of the disease. Their prolonged use is associated with severe adverse effects, including nausea, diarrhea, vomiting, dizziness, bradycardia, headache, insomnia, and liver toxicity [35,36].

A new hope for AD treatment lies in the emergence of disease-modifying agents capable of altering disease progression. In 2021, the U.S. Food and Drug Administration (FDA) approved aducanumab, and in 2023, lecanemab, which are passive immunotherapy antibodies capable of reducing amyloid β (Aβ) deposition [37,38]. Clinical research has shown that over a period of 18 months, lecanemab reduced brain amyloid burden and cognitive decline by 27% in patients compared to a placebo [38]. These new drugs represent a fundamentally new approach to disease treatment. However, there is no definitive evidence yet that removing amyloid deposits will be therapeutically beneficial for all individuals diagnosed with AD, especially for patients at more advanced stages of the disease.

Given the limitations of current medications for memory impairment therapy, including their relatively low efficacy and severe adverse effects with prolonged use, there is a critical need to develop novel agents with improved therapeutic outcomes.

For centuries, the plant kingdom has been a vital source of medicinal compounds [39,40,41,42,43,44,45]. Moreover, the use of medicinal plants appears to result in fewer side effects and interactions with drugs [46]. Due to their rich diversity of secondary phytochemical metabolites, plants have shown potential in treating various neurodegenerative diseases, including AD.

Lamiaceae is one of the largest families of flowering plants, with high expectations for its potential benefits in the field of neurodegenerative diseases. Numerous articles have discussed the neurobiological effects of various Lamiaceae species. *S. officinalis*, *M. officinalis,* and *R. officinalis* are Lamiaceae plants known in different cultures as potent memory-enhancing agents [47,48,49].

Potential in vitro anti-Alzheimer effects have been reported for *S. officinalis*, *L. an-gustifolia*, *M. piperita*, *M. officinalis*, *O. onites*, *T. vulgaris* L., *O. majorana*, *O. vulgare* L., *S. congesta*, and *D. hastata*, which are also all Lamiacea species [50]. Potential anticholinesterase activity has been reported from Nepeta, Origanum, Salvia, and Mentha—species from the genus Lavandula [51].

Our previous research with *Sideritis scardica* and *Clinopodium vulgare*, also members of the Lamiaceae family, showed an ameliorative memory effect in experimental models of dementia in rodents. They positively impact spatial working and recognition memory, affected by scopolamine [22]. Notably, the combination of *S. scardica* and *C. vulgare* demonstrated the best recognition memory-preserving effect, strong antioxidant activity, and a notable monoaminergic preservation effect in the cortex. The strongest antioxidant potential and AChE inhibitory activity of *C. vulgare* have also been demonstrated. In addition, our findings indicate that *S. scardica* and *C. vulgare* mitigate the Sco-induced downregulation of the brain-derived neurotrophic factor (BDNF) and the phosphorylation of the cAMP response element-binding protein (p-CREB) signaling pathway suggests a neuroprotective effect. The preservation and enhancement of neurotrophic factors like BDNF, which supports neuronal survival and synaptic plasticity, constitute a key focus as a new strategy for AD treatment.

Perry et al. [52] reported that a mixture of sage, rosemary, and melissa supported verbal episodic memory in healthy subjects under 63 years old. *M. officinalis* significantly improved the memory and cognitive performance of young people and AD patients between 65 and 80 years of age [48,53]. The effectiveness of *S. officinalis* in the treatment of AD and memory deficits in clinical trials has been also reported [54].

*Marrubium vulgare*, also a member of the Lamiaceae family, is commonly known as white horehound. It is widely used globally as an expectorant for cough, as well as to alleviate bloating, flatulence, and temporary loss of appetite [55]. This plant is abundant in polyphenolic compounds, such as caffeic acid, p-coumaric acid, ferulic acid, rosmarinic acid, quercetin, and apigenin [23], which are recognized for their antioxidant properties and can affect the cardiovascular, gastrointestinal, immune, and endocrine systems [56,57,58,59,60]. Additionally, recent studies have shown various pharmacological effects of *M. vulgare* on the central nervous system, including the neuroprotection of short-term memory in mice using an in vivo model of traumatic brain injury, as well as antioxidant and AChEI activities [61].

Our preceding research has shown that a water extract of *M. vulgare* effectively ameliorates spatial memory impairment in a Sco-induced experimental model of AD in rats [23]. The treatment not only decreased the AChE activity in the hippocampus by 20% but also reduced oxidative stress induced by Sco in the brain. A decrease in thiobarbituric acid-reactive substances and the restoration of antioxidant enzyme activities, such as superoxide dismutase (SOD), catalase (CAT), and glutathione peroxidase (GPx), are particularly evident in the cortex of the Sco-treated rats [23].

Building on these findings, we have embarked on a further investigation of the neuroprotective potential of *M. vulgare* against memory deficits associated with neurodegeneration. We conducted an additional in vivo study assessing its effects in a Sco-induced rat model of dementia. Our study examined the impact of the plant extract on recognition memory using the Novel Object Recognition test and monitored changes in the levels of ACh and biogenic amines, along with the expression of BDNF and pCREB in the brain cortex and hippocampus. Furthermore, we assessed the relative expression levels of *BDNF*, *CREB,* and *Bcl2* in the cortex of healthy rats using quantitative real-time PCR (qRT-PCR). This comprehensive approach aims to elucidate the therapeutic potential of *M. vulgare*, paving the way for future treatments that harness the neuroprotective properties of natural compounds.

## 2. Materials and Methods

### 2.1. Plant Material and Extract Preparation

*Marrubium vulgare* plants were obtained using micropropagation techniques and cultivated in the experimental field located in Elin Pelin (near Sofia city, at an altitude of 560 m). The aerial parts of the plants were collected in July and subsequently dried at room temperature (19–21 °C).

Dried aerial parts were ground into a fine powder using a laboratory mill. Five grams of the powder were added to 200 mL water (90 °C) and incubated for 15 min. The mixture was centrifuged at 6000× *g* and the supernatant was lyophilized for 96 h using an Alpha 1–4 LD plus laboratory freeze drier (Martin Christ Gefriertrocknungsanlagen GmbH, Osterode am Harz, Germany).

### 2.2. Determination of Marrubiin Content

Marrubiin content in the extract was determined using a slightly modified method based on the European Pharmacopoeia (Ph Eur) monograph 1835 [62]. Chromatographic analyses were conducted using a Nexera-i LC2040C Plus UHPLC system (Shimadzu Corporation, Kyoto, Japan), equipped with a UV-VIS detector and a binary pump. The analytical column employed was a Poroshell 120 EC-C18 (3 mm × 100 mm, 2.7 μm), thermostated at a temperature of 26 °C. The flow rate was set at 1.5 mL/min, with an injection volume of 20 μL. Derivatives were detected at a wavelength of 330 nm. The mobile phase consisted of component A: acetonitrile, and component B: 0.5 mL of phosphoric acid R diluted to 1000 mL with water. The gradient condition was applied as specified.
**Time, min****A (%)****B (%)**0–1540–9060–1015–2090–4010–6020–254060

The content of marrubiin in the extract, expressed in grams per 100 grams (g/100 g), was calculated using the following formula:

$$\frac{A1\times M2\times P\times 2.5}{A2\times M1}$$

where:

A1 = the area of the marrubiin in the chromatogram of the test solution.
A2 = the area of the marrubiin peak in the chromatogram of the reference solution.
M1 = the mass of the extract being examined, in milligrams.
M2 = the mass of marrubiin R, in milligrams.
P = the percentage content of marrubiin in the marrubiin standard

### 2.3. Animals

Male Wistar rats weighing 200–250 g were obtained from the animal house Erboj, Slivniza, Bulgaria. The rats were housed under standard laboratory conditions with temperatures maintained at 25 ± 3 °C and a consistent 12/12 h light/dark cycle. They had ad libitum access to a pellet diet and water. To acclimate the rats to the environment, they underwent a five-day habituation period prior to the experiment. The experimental protocol followed the guidelines of the European Communities Council Directive (86/609/EEC) and received approval from the Bulgarian Food Safety Agency (Approval No. 340/13.12.22 for working with laboratory animals).

### 2.4. Experimental Design

**Figure 1:**
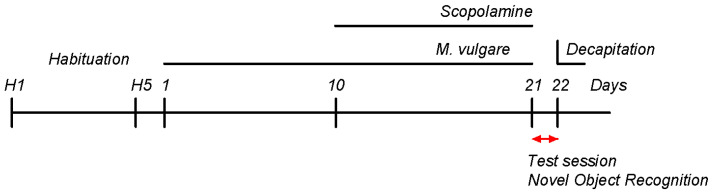
Schematic timeline of the experimental paradigm.

The animals were randomly assigned into four groups, each consisting of 12 rats: 1. Control; 2. Sco; 3. Sco + *M. vulgare*; 4. *M. vulgare*. For 21 days, the Control and Sco groups received distilled water orally (0.5 mL/100 g body weight). The *M. vulgare* and Sco + *M. vulgare* groups received an oral dosage of the plant extract at 200 mg/kg body weight for the same duration [23,56]. The Control and *M. vulgare* groups received intraperitoneal (i.p.) injections of 0.9% NaCl solution (0.5 mL/100 g body weight) for 11 consecutive days. The Sco and Sco + *M. vulgare* groups received i.p. injections of scopolamine hydrobromide (2 mg/kg) [25,63,64,65,66,67] also for 11 consecutive days to develop the experimental model of dementia. The intraperitoneal injections with Sco or 0.9% NaCl solution began 10 days after the first oral administration of the plant extract or distiller water and continued for 11 days simultaneously with the oral treatments (Figure 1).

Scopolamine was freshly dissolved in distilled water ex tempore before each administration.

### 2.5. Assessment of Cognitive Function: Novel Object Recognition Test

After completing the 21-day treatment regimen, rats from all groups underwent the Novel Object Recognition (NOR) behavioral test to evaluate their recognition memory performance (Figure 1). This behavioral assessment was conducted in a dimly lit room between 9 a.m. and 12 p.m., providing consistent environmental conditions for accurate observation of cognitive responses.

The NOR test utilizes the natural propensity of rodents to explore new objects more than familiar ones [68]. This test was conducted over two days. On the first day, a habituation session was conducted in which each rat was individually placed in an enclosed area (box measuring 60 × 60 × 60 cm) containing two identical objects, where they were allowed to explore for 3 min. The test session began 24 h after habituation and was divided into two phases, separated by a one-hour interval. In the first phase, each rat explored two identical objects in the recognition box for 4 min. An hour later, one of the original objects was replaced with a new one, and the rat was given 3 min to explore both objects. Recognition memory was assessed using the discrimination index (DI), calculated as follows: DI = (time spent on the new object/total time spent exploring both objects) × 100%. An object was considered to be explored when the animal placed its nose within 2 cm of the zone where the object was located.

### 2.6. Brain Dissection Technique

One hour after completing the test, the animals were humanely euthanized using mild CO_2_ inhalation followed by decapitation. The skin covering the skull was incised along the midline and removed to expose the dorsal skull plates. Using scissors, the plates were carefully split along the midline and then twisted outward along the lateral border to reveal the brain. The membrane enveloping the brain was delicately removed with fine forceps. The brain was then carefully removed from the skull and subjected to microdissection to isolate memory-specific regions, such as the frontal cortex and hippocampus. For hippocampus isolation, two short microspatulas were used. One spatula was positioned just above the cerebellum near its junction with the cortex, while the other gently peeled the cortical hemisphere laterally to expose the hippocampus, which was meticulously scooped out. The frontal cortex was then dissected. Throughout the procedure, the tissue was periodically rinsed with fresh ice-cold saline (0.9% NaCl) using a pipette.

### 2.7. Determination of Acetylcholine and Monoamine Content

The ACh content in tissue lysate was determined using an Enzyme-Linked Immunosorbent Assay (ELISA) kit (cat. No. E-EL-0081, Elabscience, Wuhan, China) following homogenization. The absorbance was measured using a microplate reader set to 450 nm. The results were calculated and expressed in picograms per milliliter (pg/mL).

For the determination of biogenic amines, specifically noradrenalin (NA), dopamine (DA), and serotonin (Sero), in the cortex and hippocampus of the experimental animals, the method established by Jacobowitz and Richardson [69] was employed. In brief, the extraction of NA and DA was carried out into a phosphate buffer; Sero extraction was performed using 0.1N HCl. The subsequent fluorescence reaction included the use of ethylenediaminetetraacetic acid (EDTA), an iodide solution, alkaline sulfite, and 5N CH3COOH for NA and DA. For Sero, o-phthaldehyde was used. Fluorescence intensity was then measured at specific wavelengths optimized for each monoamine: λ = 385/485 nm for NA, λ = 320/385 nm for DA, and λ = 360/470 nm for Sero. The fluorescence levels of the monoamines were calculated based on the fluorescence of the standard solution and the results are expressed in ng/g of fresh tissue.

### 2.8. Determination of BDNF and pCREB Concentrations

The concentrations of Brain-Derived Neurotrophic Factor (BDNF) and phosphorylated cAMP response element-binding (pCREB) in the frontal cortex and hippocampus of rats were quantified using ELISA. According to the manufacturer instructions, the following kits were utilized: the Rat BDNF ELISA Kit (cat. no. E-EL-R1235) from Elabscience and the Rat Phospho cAMP response element binding protein (pCREB) ELISA Kit (cat. no. SL1344Ra) from Sunlong Biotech Co., Ltd. (Hangzhou, China) Measurements were performed with a microplate reader set to 450 nm. The results were quantified and reported in picograms per milliliter (pg/mL).

### 2.9. RNA Extraction and Reverse Transcription

RNA was isolated from the cortex using the PRImeZOL reagent (Canvax), following the manufacturer’s protocol. The purity and concentration of the extracted RNA were assessed using a NanoDrop 2000 spectrophotometer (Thermo Scientific, Waltham, MA, USA). To eliminate DNA contamination, the RNA samples were treated with DNase I (Thermo Scientific). Reverse transcription was carried out using the FIREScript RT cDNA Synthesis Mix, which included oligo (dT) and random primers (Solis BioDyne, Tartu, Estonia), according to the instructions provided by the manufacturer.

### 2.10. Quantitative Real-Time PCR

The genes *BDNF*, *CREB,* and *Bcl2* were analyzed using HOT FIREPol EvaGreen qPCR Mix Plus (Solis BioDyne). The RT-PCR reactions were performed in duplicate on a 96-well plate using a PikoReal 96-RT PCR system (Thermo Scientific). The thermal cycling conditions consisted of initial denaturation at 95 °C for 12 min, followed by 35 cycles consisting of 15 s at 95 °C, 30 s at annealing temperatures of 59 °C or 58 °C, and 30 s at 72 °C. The primer sequences and their corresponding product sizes are detailed in Table 1. *GAPDH* was used as the reference gene for the normalization of the qRT-PCR data. The primer sequences for *BDNF* and *CREB* were based on Tancheva et al. [25], while those for *Bcl2* and *GAPDH* were sourced from Ramezani et al. [70] and Kharroubi et al. [71], respectively. The Relative Expression Software Tool V2.0.13 (REST) [72] facilitated the group comparison and statistical analysis of relative expression results obtained from RT-PCR experiments.

### 2.11. Statistical Analysis

Data are presented as the mean ± standard error of the mean (SEM). Analyses were performed with Prism 8.0 software (GraphPad Software, Inc., San Diego, CA, USA). Statistical testing involved one-way analysis of variance (ANOVA) followed by Tukey’s post hoc comparison test to determine significant differences between groups. A *p*-value of less than 0.05 (*p* < 0.05) was considered statistically significant.

## 3. Results

### 3.1. Effects of M. vulgare Treatment on Recognition Memory Performance in Healthy and Scopolamine-Treated Rats

**Figure 2:**
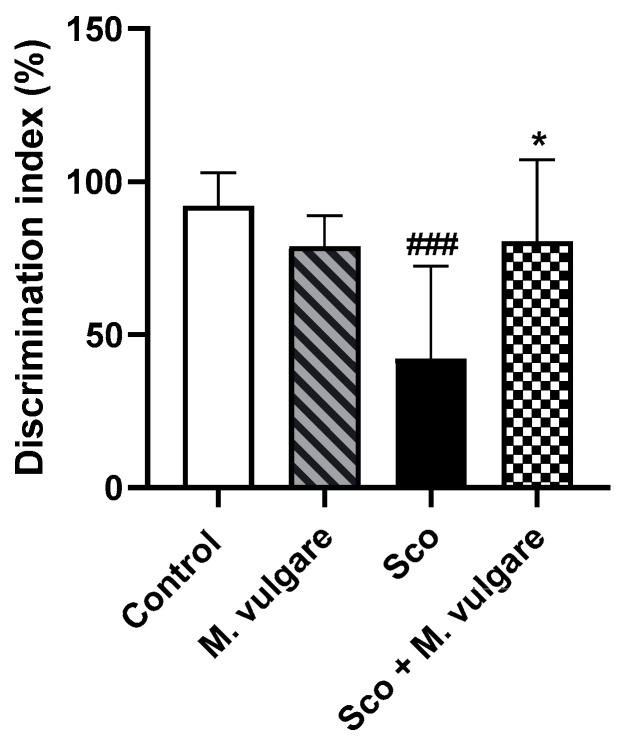
Effect of *M. vulgare* extract on cognitive performance of healthy rats and those with scopolamine-induced memory impairment. Recognition memory was evaluated through the Novel Object Recognition test after a 21-day administration of *M. vulgare* extract. Data are presented as mean values ± standard error of the mean (SEM) for groups of 12 animals each. Statistical significance is indicated as follows: ### *p* < 0.001 compared to the saline-treated group; * *p* < 0.05 compared to the scopolamine-treated group.

In this study, we evaluated recognition memory performance using the Discrimination Index (DI) as illustrated in Figure 2. Analysis of the results showed that 11 days of Sco treatment significantly impaired recognition memory with a 54.29% decrease in DI (*p* < 0.001, *n* = 6). This indicated that the Sco-treated rats spent significantly less time interacting with the new object compared to the control group. After 21 days, treatment with *M. vulgare* effectively reversed the memory deficits induced by Sco. The value of DI in the Sco + *M. vulgare* group increased by 91.10% compared to the untreated Sco group. In healthy rats treated with *M. vulgare*, there was no significant change in DI compared to the saline-treated group.

### 3.2. Neuromodulatory Activity of M. vulgare in the Frontal Cortex and Hippocampus of Healthy and Scopolamine-Treated Rats

**Figure 3:**
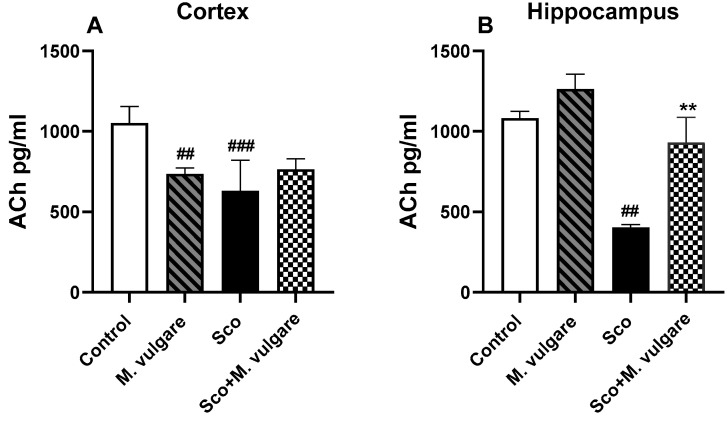
Effect of *M. vulgare* extract on acetylcholine levels in the cortex (**A**) and hippocampus (**B**) of healthy rats and those with scopolamine-induced memory impairment. Acetylcholine levels were evaluated after a 21-day administration of *M. vulgare* extract. Data are presented as mean values ± SEM (*n* = 6 animals per group). Statistical significance is indicated as follows: ### *p* < 0.001, ## *p* < 0.01 compared to the saline-treated group; ** *p* < 0.01 compared to the scopolamine-treated group.

**Figure 4:**
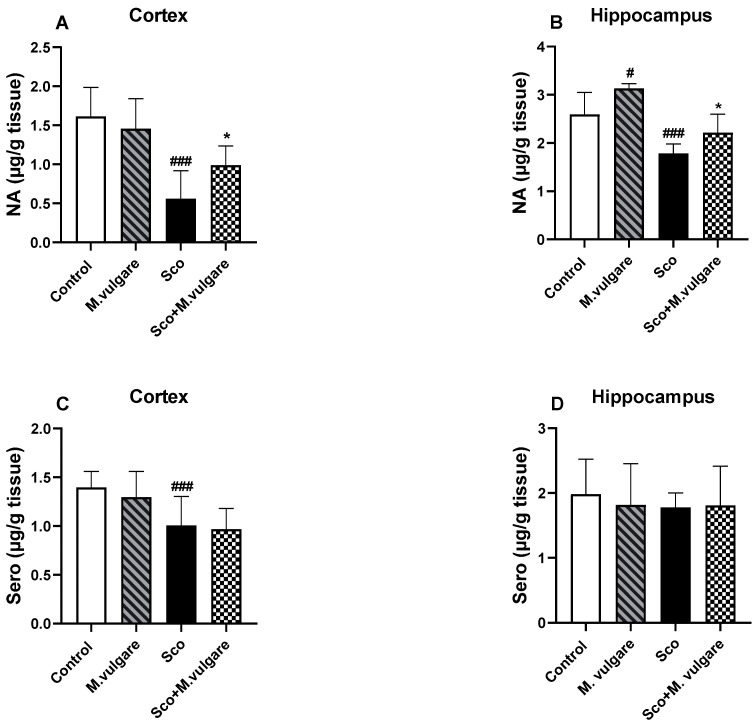
Effect of *M. vulgare* extract on the content of brain biogenic amines in healthy rats and those with scopolamine-induced memory impairment. The content of noradrenaline (**A**,**B**) and serotonin (**C**,**D**) was assessed in the cortex and hippocampus of rats after a 21-day administration of *M. vulgare* extract. Data are presented as mean values ± SEM (*n* = 6 animals per group). Statistical significance is indicated as follows: # *p* < 0.05, ### *p* < 0.001 compared to the saline-treated group; * *p* < 0.05 compared to the scopolamine-treated group.

The neuromodulatory effects of *M. vulgare* extract were evaluated by measuring the levels of ACh, noradrenaline (NA) and Sero in the cortex and hippocampus of rats from all experimental groups (Figure 3A,B and Figure 4A–D). Our findings revealed that Sco treatment significantly decreased the levels of ACh and monoamines in rats with dementia-like symptoms. In the cortex of these animals, ACh levels decreased by 39.96% (*p* < 0.001, *n* = 6), NA by 65.26% (*p* < 0.001, *n* = 6), and Sero by 28.08% (*p* < 0.001, *n* = 6), compared to the control group. In the hippocampus, ACh and NA levels decreased by 62.73% (*p* < 0.01, *n* = 6) and 31.12% (*p* < 0.001, *n* = 6), respectively, compared to the saline-treated rats. However, Sero levels in the hippocampus of Sco-treated animals did not show significant changes following 21 days of *M. vulgare* treatment (Figure 4D).

Treatment with *M. vulgare* for 21 days demonstrated a protective effect on ACh and NA levels and did not significantly alter Sero levels in the brains of Sco-treated rats. In the cortex, the plant extract increased NA levels by 76.33% (*p* < 0.05, *n* = 6) compared to Sco-treated rats. In the hippocampus, ACh content was increased by 130.89% (*p* < 0.01, *n* = 6) and NA levels were enhanced by 23.91% (*p* < 0.05, *n* = 6) compared to the dementia-like animals.

In healthy rats, *M. vulgare* treatment had a significant impact only on ACh and NA levels in the brain. In the cortex, ACh levels were decreased by 30.11% (*p* < 0.01, *n* = 6), while NA and Sero levels remained largely unaffected compared to the saline-treated animals. In the hippocampus, the plant extract showed a trend toward increasing ACh content (although not significantly) and raised NA levels by 20.57% (*p* < 0.05, *n* = 6), without significant changes in Sero levels compared to controls.

### 3.3. Effect of M. vulgare on the Expression Levels of BDNF and pCREB in the Frontal Cortex and Hippocampus of Healthy and Scopolamine-Treated Rats

**Figure 5:**
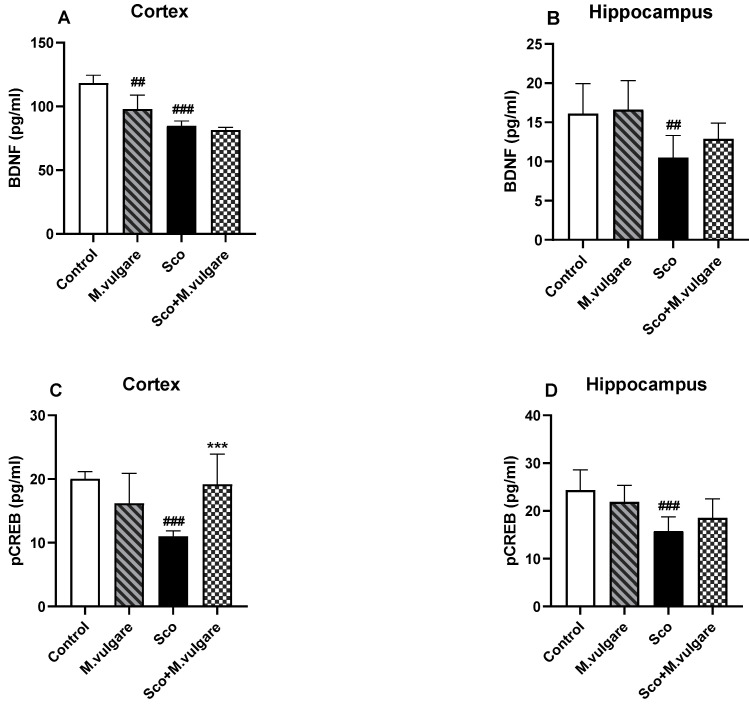
Effect of *M. vulgare* extract on the expression of BDNF and pCREB in the brains of healthy rats and those with scopolamine-induced memory impairment. The concentrations of BDNF and pCREB were assessed using the Enzyme-Linked Immunosorbent Assay (ELISA) in the rat cortex and hippocampus after a 21-day administration of *M. vulgare* extract. (**A**) BDNF levels in the cortex. (**B**) BDNF levels in the hippocampus. (**C**) pCREB levels in the cortex. (**D**) pCREB levels in the hippocampus. Data are presented as mean values ± SEM (*n* = 6 animals per group). Statistical significance is indicated as follows: ## *p* < 0.01; ### *p* < 0.001 compared to the saline-treated group; *** *p* < 0.001 compared to the scopolamine-treated group.

The expression levels of BDNF and pCREB in the cerebral cortex and hippocampus were evaluated by the ELISA method. According to our results, 11 days of Sco administration significantly decreased the expression of BDNF and pCREB in these brain regions (Figure 5A–D). Specifically, in the cortex, BDNF expression was reduced by 28.44% (*p* < 0.001, *n* = 6) and pCREB by 45.24% (*p* < 0.001, *n* = 6) (Figure 5A,C). In the hippocampus, BDNF expression decreased by 34.98% (*p* < 0.01, *n* = 6) and pCREB by 35.15% (*p* < 0.001, *n* = 6) (Figure 5B,D). These comparisons were made against saline-treated rats.

Treatment with *M. vulgare* for 21 days significantly counteracted the Sco-induced changes in pCREB expression in the cortex. Specifically, pCREB levels increased by 74.77% (*p* < 0.001, *n* = 6) compared to the untreated Sco group.

In healthy animals, ELISA analysis indicated that the plant extract treatment generally did not result in significant changes in pCREB/BDNF signaling in the frontal cortex and hippocampus. The sole exception was observed in the cortex, where BDNF expression decreased by 17.26% (*p* < 0.01, *n* = 6) compared to the control group.

### 3.4. Effect of M. vulgare Treatment on Relative Expression Levels of BDNF, CREB, and Bcl2 in the Cortex of Healthy Rats

**Figure 6:**
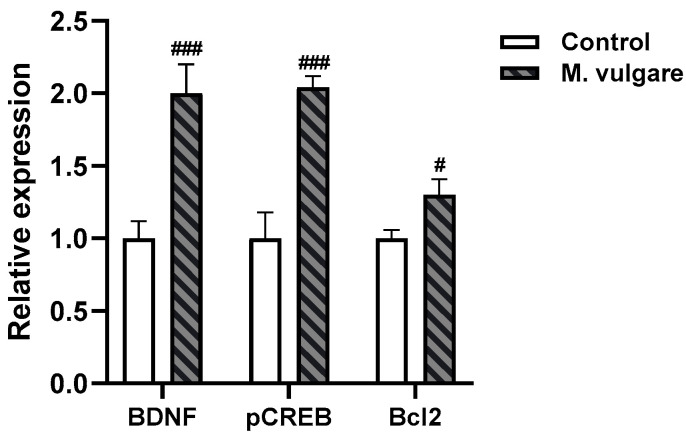
Relative expression levels of BDNF, CREB, and Bcl2 in the cortex of healthy rats. Gene expression was analyzed using qRT-PCR after a 21-day administration of *M. vulgare* extract. Data represented as mean values ± SEM (*n* = 10 animals per group). Statistical significance is indicated as follows: # *p* < 0.05, ### *p* < 0.001 compared to the saline-treated group.

We employed an RT-PCR assay to investigate potential differences in the expression of the BDNF, CREB, and Bcl2 genes between *M. vulgare*-treated and control rats (Figure 6). Our results revealed significant upregulation in the expression of all three genes. Specifically, BDNF expression increased to 2.0±0.20 (*p* < 0.001, *n* = 10), while CREB and Bcl2 expressions rose to 2.04 ± 0.08 (*p* < 0.001, *n* = 10) and 1.3 ± 0.11 (*p* < 0.05, n = 10), respectively, compared to the control group.

## 4. Discussion

In this research, we demonstrated that treatment with *M. vulgare* extracts substantially improved recognition memory in rats with Sco-induced cognitive deficits. A 21-day oral administration of the extract appeared to stabilize the cholinergic system, as evidenced by elevated levels of ACh in the hippocampus. Moreover, our findings suggest that the plant extract also impacted the NA system and key signaling molecules involved in memory formation and neural plasticity, namely BDNF and CREB. These observations support our previous research, which demonstrated that a water extract of *M. vulgare* improved spatial memory in a dementia rat model through mechanisms involving antioxidant and AChEI activities [23]. These results not only affirm the neuroprotective properties of *M. vulgare* but also highlight its potential to counteract the biochemical pathways of memory decline typically observed in Sco-induced models of dementia.

Sco is a muscarinic antagonist known to induce cognitive deficits in healthy humans and rodents by blocking cholinergic signaling [18]. Its intraperitoneal administration is widely accepted as a pharmacological model of AD in rodents, aligning with the cholinergic hypothesis. This hypothesis suggests that defects in the central cholinergic system or alterations in hippocampal function in the brain are implicated in human memory disorders [73]. The administration of Sco altered the behavior and biochemical profiles of the experimental animals. Deficiency in spatial memory, associative memory, and recognition memory performance are reported in our studies and by other researchers [20,21,22,23,24,25,64]. In addition to disrupting the brain cholinergic system (evidenced by increased AChE activity and reduced ACh levels), Sco treatment also escalated oxidative stress, diminished brain monoamine levels (DA, NA, and Sero), and attenuated BDNF/CREB signaling in the brain [22,23,24,25,63,74,75,76].

In this study, we employed the NOR test to evaluate the effect of the *M. vulgare* treatment on Sco-induced memory impairment in rats. The NOR test is a widely used method for evaluating cognitive deficits in experimental animals, capitalizing on the innate inclination of rodents to explore new objects more than familiar ones. This task incorporates elements of exploratory behavior and memory retention, requiring that animals adequately explore the familiar object during the pretest phase to enable them to differentiate it from a novel object later in the test phase [77]. Animals with preserved memory formation tend to spend more time with the new object during the actual test. Our findings show that rats treated with Sco displayed reduced exploration of novel objects compared to controls. This aligns with our previous observations, where Sco treatment significantly lowers the discrimination index in the NOR task and decreases the exploratory behavior of animals in other behavioral tests like the Hole board test [22,25,64].

Our analyses showed that 21 days of *M. vulgare* treatment did not significantly change recognition memory in healthy rats versus controls; however, it effectively mitigated memory impairment in rats with Sco-induced cognitive decline. These results indicate that the extract sustained memory formation under conditions of cognitive stress. This is consistent with our previous research, which demonstrated that *M. vulgare* preserved spatial memory against Sco-induced damage, as evidenced in the T-maze test. Moreover, we have verified that the memory-related effects of Sco and *M. vulgare* are not due to changes in motor function or sedation of the experimental animals, underscoring the specific cognitive benefits of the plant extract treatment [23].

The ameliorated memory effects of the extract may be attributed to the diversity of its secondary phytochemical metabolites. Our analysis revealed that *M. vulgare* extract is rich in polyphenolic compounds, such as caffeic acid, p-coumaric acid, ferulic acid, rosmarinic acid, quercetin, and apigenin [23]. Marrubiin, a well-known diterpenoid lactone prevalent in horehound and other medicinal plants of the Lamiaceae family, was present in the extract standardized to 2.5% marrubiin content. Marrubiin is recognized for its broad pharmacological properties, including high safety, low turnover, high stability, and minimal catabolism. Additionally, it has revealed a range of important biological activities including antinociceptive, antigenotoxic, cardioprotective, vasorelaxant, gastroprotective, antispasmodic, immunomodulating, antioedematogenic, analgesic, and antidiabetic properties [78]. The neuroprotective potential of marrubiin following traumatic brain injury in experimental mice [61] and potent AChEI activity [79] have also been reported and could explain the results of our study. Furthermore, literature data indicate that rosmarinic acid, another phenolic constituent found in horehound, has a beneficial effect on Sco-induced learning and memory impairment in rat models [80]. In addition, apigenin has been reported to improve cognitive dysfunction and neuroinflammation by upregulating the ERK/CREB pathway in APP/PS1 transgenic AD mice [81]. These findings collectively provide a comprehensive explanation for the cognitive benefits observed in our study with *M. vulgare* extract.

To identify the specific components of cholinergic neurotransmission affected by *M. vulgare* treatment, we measured ACh levels in the cortex and hippocampus of healthy and dementia rats. This analysis complements our prior findings, which highlighted the AChEI activity of *M. vulgare*, particularly noting its pronounced effects in the hippocampus [23]. ACh, a key component of the cholinergic system, has been a focal point of memory research over the last decade. It is well documented that fluctuations in ACh levels or AChE activity can disrupt cholinergic transmission, resulting in learning and memory deficits similar to those seen in AD [82]. Therefore, assessing acetylcholinesterase activity and ACh levels in the brain can provide valuable information on cholinergic function, which is closely connected to cognitive performance.

Our results clearly indicate that Sco administration to experimental animals led to a significant reduction in brain ACh levels compared to the control group. The subsequent administration of the plant extract for three weeks elevated ACh levels in the hippocampus of rats suffering from Sco-induced memory impairment. Consequently, we could suggest that the positive effects of *M. vulgare* extract and its major diterpene component, marrubiin, in mitigating Sco-induced memory deficits may be linked to increased central cholinergic functioning.

The brain monoaminergic system is another key factor in memory-related processes. NA, a component of this system, is essential for the formation of hippocampus-based declarative memory [83].

The locus coeruleus (LC) is the primary source of NA in the central nervous system and plays a crucial role in memory formation by projecting to key brain areas, such as the hippocampus [84], amygdala [85], and prefrontal cortex [86]. It is noteworthy that the LC is activated in response to novelty [87] and arousal [88], and its degeneration is a hallmark of AD [89]. Sco treatment has been shown to depress the monoaminergic systems in the cortex and hippocampus, a finding corroborated by our previous research [22,25,64]. Treatment with *M. vulgare* has been observed to positively affect the NA system but did not significantly impact the Sero system.

There are data indicating a close correlation between reduced expression of BDNF and CREB in the hippocampus and cognitive impairments. This suggests that the transcription of BDNF, which is regulated by CREB, plays a critical role in the adaptive neuronal responses that underpin learning and memory functions [90,91].

Traditionally recognized as a neurotrophic factor essential for neuronal cell survival and the prevention of neurodegeneration, BDNF also plays a pivotal role in modulating synaptic function and plasticity in the central nervous system, particularly in learning and memory processes [92]. CREB, a transcription factor, is instrumental in regulating the expression of genes involved in neuroplasticity, cell survival, and cognition [93]. Phosphorylated CREBs can bind to the cAMP response elements of target genes associated with long-term memory formation [94]. BDNF is a prominent target of CREB signaling [95,96], indicating that BDNF transcription, regulated by CREB, is vital in the adaptive neuronal responses that support learning and memory function [90].

In the current study, the expression levels of BDNF and pCREB in the hippocampus and frontal cortex of the animals were evaluated by the ELISA method. Analysis revealed that, as expected, Sco suppressed hippocampal and cortical pCREB/BDNF protein expression, leading to memory impairment in rats. This aligns with literature data and findings from our previous research [22,25,97]. Treatment with *M. vulgare* significantly increased pCREB expression in the cortex of the animals with dementia. In the hippocampus, there was only a tendency for an ameliorative effect of the extract on suppressed BDND/pCREB signaling by Sco.

In the healthy animals, *M. vulgare* treatment led to complex neurochemical alterations: after 21 days of administration, there was a decrease in ACh levels and BDNF expression in the cortex, while NA levels in the hippocampus increased. Interestingly, the gene expression of the neurotrophic factor *BDNF* in the cortex of healthy rats was elevated post-treatment. In addition, in our genetic studies, we also observed significant changes in the expression of the cellular transcription factor *CREB* and the antiapoptotic factor *Bcl2*.

Despite the promising results on the neuroprotective activity of *M. vulgare* extract, this study has limitations, including the absence of histopathological evaluations of the frontal cortex and hippocampus. Future research will focus on addressing these gaps. Considering the safety profile and demonstrated benefits of *M. vulgare*, its integration into existing treatment paradigms for cognitive impairment could improve therapeutic outcomes and enhance the quality of life for patients with neurodegenerative conditions.

## 5. Conclusions

Our research demonstrated the memory-protective effects of *M. vulgare* in a Sco-induced model of dementia, evaluated using the NOR test. The observed benefits on memory appear to be linked to several neural mechanisms: the modulation of cholinergic function in the hippocampus, the regulation of NA neurotransmission in the brain, and alterations in the gene and peptide expression of key memory-related molecules such as BDNF and CREB. These findings suggest that *M. vulgare* could influence critical pathways involved in memory formation and retention. Given these results, there is a compelling need for more in-depth studies to explore the mechanisms of action of *M. vulgare* extract. Expanding this line of inquiry could further delineate its mechanisms of action and reinforce its potential as a therapeutic agent for cognitive impairments in various neurodegenerative diseases.

## Figures and Tables

**Table 1:** Primer sequences, annealing temperatures, and product sizes for genes analyzed in Real-Time PCR.

Gene.	Primer sequences (5′-3′)	Annealing Temperature (°C)	Product Size (bp)
*BDNF*	TGGCTGACACTTTTGAGCAC	58	188
	GTTTGCGGCATCCAGGTAAT		
*CREB*	TGCACAGACCACTGATGGAC	59	286
	TTCAAGCACTGCCACTCTGT		
*Bcl2*	GGATTGTGGCCTTCTTTGAGTTC	59	88
	AGAGCGATGTTGTCCACCAG		
*GAPDH*	ACCACAGTCCATGCCATCACTGCCAC	58	447
	TCCACCACCCTGTTGCTGTA		

## Data Availability

All data are contained in the manuscript.
